# Reliability and validity of the Roche PD Mobile Application for remote monitoring of early Parkinson’s disease

**DOI:** 10.1038/s41598-022-15874-4

**Published:** 2022-07-15

**Authors:** Florian Lipsmeier, Kirsten I. Taylor, Ronald B. Postuma, Ekaterina Volkova-Volkmar, Timothy Kilchenmann, Brit Mollenhauer, Atieh Bamdadian, Werner L. Popp, Wei-Yi Cheng, Yan-Ping Zhang, Detlef Wolf, Jens Schjodt-Eriksen, Anne Boulay, Hanno Svoboda, Wagner Zago, Gennaro Pagano, Michael Lindemann

**Affiliations:** 1 Roche Pharma Research and Early Development, pRED Informatics, Pharmaceutical Sciences, Clinical Pharmacology, and Neuroscience and Rare Diseases Discovery and Translational Area, Roche Innovation Center Basel, F. Hoffmann-La Roche Ltd., Grenzacherstrasse 124, 4070 Basel, Switzerland; 2 Department of Neurology, McGill University, Montreal General Hospital, Montreal, QC Canada; 3 Paracelsus-Elena-Klinik, Kassel, Germany; 4 Department of Neurology, University Medical Center Göttingen, Göttingen, Germany; 5 Idorsia Pharmaceuticals Ltd, Allschwil, Switzerland; 6 Prothena Biosciences Inc, South San Francisco, CA USA

**Keywords:** Parkinson's disease, Neurological disorders, Biomarkers

## Abstract

Digital health technologies enable remote and therefore frequent measurement of motor signs, potentially providing reliable and valid estimates of motor sign severity and progression in Parkinson’s disease (PD). The Roche PD Mobile Application v2 was developed to measure bradykinesia, bradyphrenia and speech, tremor, gait and balance. It comprises 10 smartphone active tests (with ½ tests administered daily), as well as daily passive monitoring via a smartphone and smartwatch. It was studied in 316 early-stage PD participants who performed daily active tests at home then carried a smartphone and wore a smartwatch throughout the day for passive monitoring (study NCT03100149). Here, we report baseline data. Adherence was excellent (96.29%). All pre-specified sensor features exhibited good-to-excellent test–retest reliability (median intraclass correlation coefficient = 0.9), and correlated with corresponding Movement Disorder Society–Unified Parkinson's Disease Rating Scale items (rho: 0.12–0.71). These findings demonstrate the preliminary reliability and validity of remote at-home quantification of motor sign severity with the Roche PD Mobile Application v2 in individuals with early PD.

## Introduction

Clinical and drug development research in Parkinson’s disease (PD) critically requires the precise quantification of clinical disease severity and its progression over time. This is a challenge in PD because of the fluctuating and slowly progressive nature of the disease. Moreover, the development of disease-modifying therapies focus on the earliest possible point in the course of the disease, when the least amount of neurodegeneration has taken place, and when disease severity and progression may be even more subtle^1^. Digital health technologies (DHTs)^2^, such as smartphones and smartwatches, may aid in overcoming these challenges, since they enable remote and therefore frequent measurement of motor signs to provide potentially more robust—i.e. reliable and valid—quantification of disease severity and its changes over time^3–5^. Moreover, the inertial measurement unit sensors (e.g. accelerometers, gyroscopes) are highly sensitive to minute changes (even in consumer technologies) and therefore may detect motor changes not evident upon routine clinical examination. While not less important, non-motor signs remain largely inaccessible to sensor measurements via DHTs. Here, we describe a novel DHT, the Roche PD Mobile Application v2, which was designed to measure motor manifestations in early PD, and demonstrate its test–retest reliability and validity in a group of de novo diagnosed individuals with PD.

Clinical trials of future potentially disease-modifying therapies for PD^6^ are especially promising among individuals who have been recently diagnosed, when less neurodegeneration has occurred. Clearly, to detect potential treatment benefit, sensitive measures of changes in motor signs in the earliest stages of the disease are required. In the Parkinson’s Progression Markers Initiative (PPMI) cohort^7^, Rasch Measurement Theory (RMT) analyses of the Movement Disorder Society–Unified Parkinson's Disease Rating Scale (MDS-UPDRS)^8^ Part II and III scores at screening, 12-month and 24-month visits (n = 384) were applied to explore potential biases in the ordinal MDS-UPDRS items comprising the subscale scores^9^. These revealed an apparent staged order of motor sign progression from unilateral bradykinesia and rigidity (first upper then lower extremities) to midline functions to bilateral bradykinesia and rigidity, and finally general movement problems^10^. These findings confirm the centrality of bradykinesia in early PD^11^ and suggest that bradykinesia is a critical motor progression marker in early PD^1^. However, the quantification of bradykinesia and other motor signs in early PD with rating scales such as the MDS-UPDRS remains a challenge: MDS–UPDRS Part II scores change little in early PD^12^, i.e. less than the established minimal clinically meaningful difference^13^, and RMT analyses of MDS-UPDRS Part III item scores revealed multiple measurement irregularities in scores during the first 2 years of PPMI, including floor effects, item gaps for very mild motor signs and item misfits (i.e. items that did not fit the ordered progression pattern)^10,14^. These findings highlight the urgent need for alternative methods of motor sign quantification in the earliest stages of PD.

Many smartphone- and smartwatch-based DHTs have been developed to estimate bradykinesia and other motor signs of PD in research settings, including the present DHT solution^5,15–21^. The individual tasks and measurements are comparable, as described in the Discussion section. With respect to early PD, finger tapping is one of the most commonly used DHT measures of bradykinesia^21^. When sensor-based finger-tapping data are aggregated over 2-week periods, test–retest reliabilities increase^5^ and correlate with MDS-UPDRS Part III clinician ratings of finger-tapping performance in patients with early PD^5^. Additional bradykinesia tests implemented on smartphones include measures of hand turning and leg agility (by holding the phone on the thigh and lifting and stomping the foot), for example as implemented on CloudUPDRS^16,17^. Smartwatches offer additional means to estimate bradykinesia during daily life, for example, by estimating the time taken to move a utensil from a plate to the mouth while eating^15^. Most DHT solutions for PD such as CloudUPDRS^16,17^, HopkinsPD^18^, mPower^19^ and the Roche PD Mobile Application v1^5^ test not only bradykinesia, but also tremor, gait, and balance, thereby providing a profile of motor impairments for estimating and tracking PD motor severity. DHTs may additionally benefit from measures of cognition such as information processing speed (e.g. electronic Symbol Digit Modalities Test [eSDMT]) and speech, which are also affected in the earliest stages of PD^20^.

The present report describes the reliability and validity of the Roche PD Mobile Application v2, a revision of v1 to include multiple novel measures of bradykinesia and cognition (eSDMT and speech tests), and to optimize existing tasks for the detection of PD motor signs. Three hundred and sixteen individuals with early-stage PD (< 2 years; Hoehn and Yahr Stages I or II) who are participating in the Phase II PASADENA study (NCT03100149) were provided with a smartphone and smartwatch with the Roche PD Mobile Application v2 preinstalled, and requested to perform active tests daily on the smartphone (4 or 5 out of 10 tests each day, information processing speed once per fortnight), and to carry the smartphone and wear a smartwatch throughout the day to collect passive monitoring data. Pre-specified sensor features were calculated for each active test and for passive monitoring, and aggregated over the first two 2-week periods of the study. Adherence, test–retest reliability, and clinical validity (relationship to baseline clinical scales, known-groups validity) were quantified. These metrics represent the grounds for judging the potential utility of the Roche PD Mobile Application v2 to quantify and track progression of disease severity in early PD.

## Results

### Adherence

On average, daily remote active testing took a median of 5.3 (interquartile range [IQR] = 1.7) minutes on days without the SDMT, and 7.32 (median; IQR = 1.58) minutes on days with SDMT. Average adherence was high with 96.29% (median per participant) of all possible active tests performed during the first 4 weeks of the study (i.e. 26–27/27 days). Participants contributed a median of 8.6 h/day of study smartphone and a median of 12.79 h/day of study smartwatch passive monitoring data.

### Reliability of sensor features

Reliability results are reported in Table 1. All 17 pre-specified sensor features demonstrated good-to-excellent^22^ test–retest reliability between the first two 2-week study periods (intraclass correlation coefficient [ICCs] ≥ 0.75) (Table 1)^23^. The median sensor feature ICC was 0.9 (range, 0.75–0.95).

**Table 1:** Reliability and clinical validity of pre-specified Roche PD Mobile Application v2 active test and passive monitoring sensor features.

Digital test	Sensor feature	MDS-UPDRS item	Test–retest reliability	Spearman’s correlation coefficient		MDS-UPDRS item score 0 versus 1					Hoehn and Yahr Stage I versus II				
			ICC (95% CI)	*r*_s_	*P* value	N	U stat	*P* value	N 0/N 1	Percent difference on group medians	U stat	*P* value	N 1/N 2	Percent difference on group medians	Feature differences in expected direction
Draw A Shape	Spiral celerity (L)	3.5 Hand movement (L)	0.91 (0.88–0.93)	− 0.15	0.0092	300	7061	0.0043	184/95	− 14.06	6681	0.0047	74/226	− 9.5	Yes
	Spiral celerity (M)	3.5 Hand movement (M)	0.92 (0.89–0.94)	− 0.12	0.0334	300	3091	0.0981	49/144	− 14.29	6463	0.0017	74/226	− 15.2	Yes
Dexterity	Tapping variability (L)	3.4 Finger tapping (L)	0.84 (0.79–0.88)	0.32	< 0.0001	300	6786	0.0006	152/116	27.33	6444	0.0015	74/226	27.16	Yes
	Tapping variability (M)	3.4 Finger tapping (M)	0.88 (0.84–0.91)	0.37	< 0.0001	300	1261	0.0006	30/135	40.64	6691	0.005	74/226	17.95	Yes
Hand Turning	Median Hand Turning speed (L)	3.6 Pronation-supination (L)	0.92 (0.89–0.94)	− 0.18	0.0015	300	7930	0.0361	192/95	− 7.85	7268	0.0458	74/226	− 7.18	Yes
	Median Hand Turning speed (M)	3.6 Pronation-supination (M)	0.95 (0.93–0.97)	− 0.46	< 0.0001	300	1912	0.0008	43/131	− 12.37	7216	0.0385	74/226	− 9.33	Yes
Speech	MFCC2 variability	3.1 Speech	0.77 (0.69–0.83)	− 0.29	< 0.0001	295	5692	< 0.0001	126/138	− 13.68	6395	0.0412	71/209	− 4.47	Yes
Phonation	Voice jitter	3.1 Speech	0.88 (0.85–0.91)	0.18	0.0017	297	7381	0.0001	135/146	21.65	7248	0.073	73/224	12.87	Yes
Postural Tremor	Log median squared energy (L)	3.15 Postural tremor (L)	0.83 (0.77–0.87)	0.23	< 0.0001	300	3240	0.0002	259/39	8.26	7111	0.0343	73/227	1.2	Yes
	Log median squared energy (M)	3.1 Postural tremor (M)	0.92 (0.90–0.94)	0.5	< 0.0001	300	4779	< 0.0001	150/116	12.45	8108	0.3918	73/227	1.28	Yes
Rest Tremor	Log median squared energy (L)	3.17 Rest tremor amplitude (L)	0.91 (0.88–0.93)	0.34	< 0.0001	300	967	< 0.0001	274/19	23.73	6614	0.0048	73/227	6.12	Yes
	Log median squared energy (M)	3.17 Rest tremor amplitude (M)	0.94 (0.91–0.95)	0.71	< 0.0001	300	1565	< 0.0001	124/71	19.27	7896	0.2731	73/227	3.59	Yes
Balance	Log sway jerk	3.12 Postural stability	0.82 (0.76–0.87)	0.14	0.0138	299	890	0.0071	287/11	10.6	7988	0.3870	72/227	1.29	Yes
U-turn	Median turn sp eed	3.14 Body bradykinesia	0.91 (0.88–0.93)	− 0.22	0.0001	299	3113	0.0539	44/168	− 5.39	6322	0.0019	72/227	− 7.83	Yes
eSDMT	Number of correct responses	1.1 Cognitive impairment	0.75 (0.66–0.81)	− 0.18	0.0014	303	3787.5	0.0012	248/43	− 10	6538	0.0008	76/227	− 7.14	Yes
Passive monitoring of gait	Median turn speed	3.14 Body bradykinesia	0.82 (0.77–0.89)	− 0.22	0.0001	285	3130	0.154	43/162	− 3.3	6270	0.0138	71/214	− 4.65	Yes
Passive monitoring of non-gait arm movements	Median arm movement power (non-gait)	3.6 Pronation-supination, left hand	0.85 (0.80–0.89)	− 0.43	< 0.0001	269	3108	< 0.0001	106/89	− 35.73	5221	0.0242	60/209	− 30.64	Yes

“M” = most affected side; “L” = least affected side.

*CI* confidence interval, *eSDMT* electronic Symbol Digit Modalities Test, *ICC* intraclass correlation coefficient, *L* less affected side, *M* more affected side, *MDS-UPDRS* Movement Disorder Society—Unified Parkinson's Disease Rating Scale, *MFCC2* Mel Frequency Cepstral Coefficient 2.

### Clinical validity of sensor features

**Figure 1:**
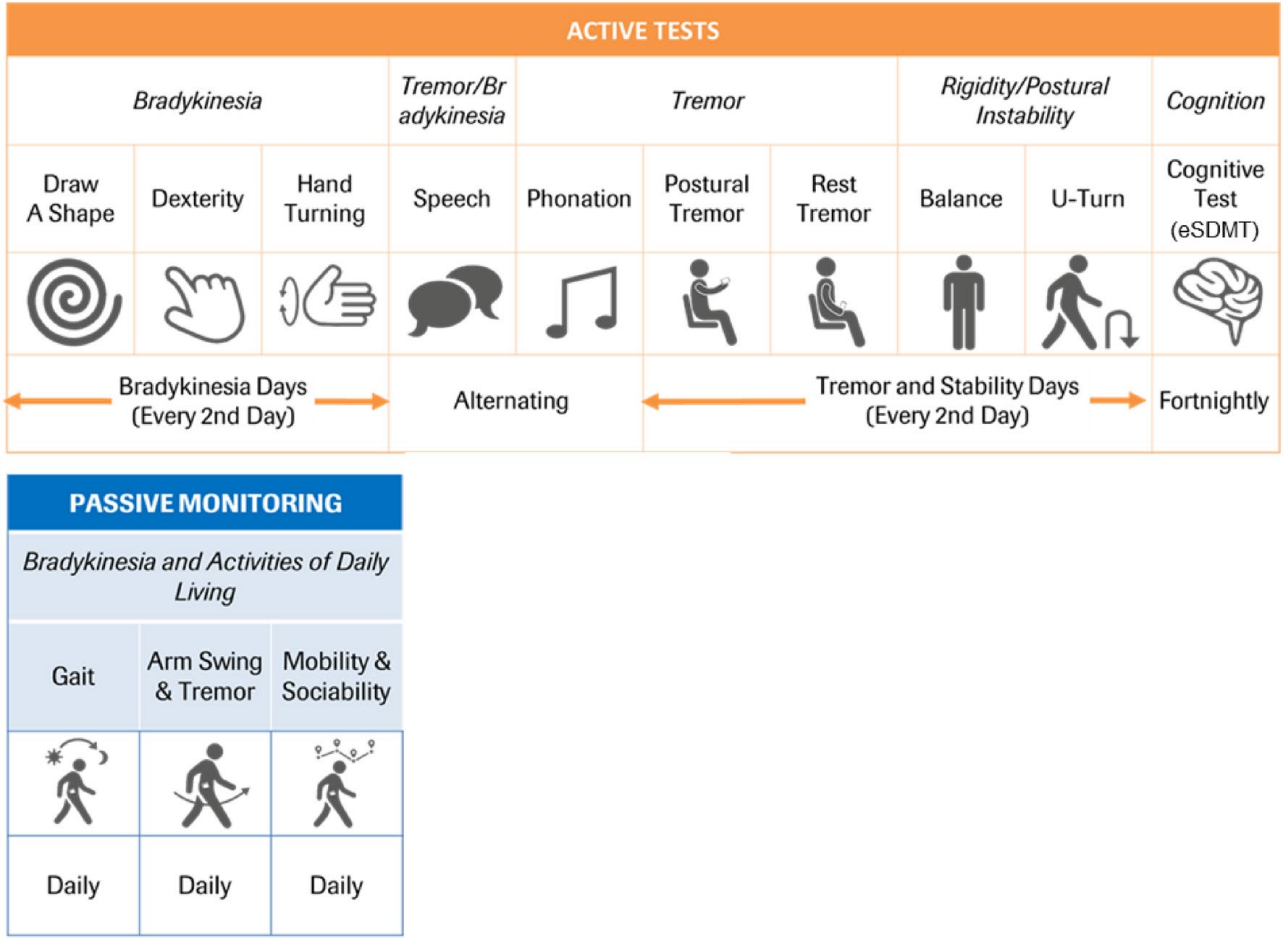
Roche PD Mobile Application v2 active tests and passive monitoring and schedule of assessments. eSDMT, electronic Symbol Digit Modalities Test; PD, Parkinson’s disease.

Clinical validity was assessed via Spearman’s correlations between the sensor features and corresponding MDS-UPDRS subscale and item scores (Fig. 1). Correlations with MDS-UPDRS item scores revealed that all sensor features correlated with their corresponding clinical items (Table 1 and Figs. 3, 4, 5). The numerically strongest relationships were observed with bradykinesia sensor features (i.e. Hand Turning and Finger Tapping) as well as postural and rest tremor sensor features, particularly for the more affected side, while the numerically weakest correlations were found with the Balance (comparator: MDS-UPDRS item 3.12 Postural stability) and Draw A Shape (comparator: MDS-UPDRS item 3.5 Hand movement) tests.

**Figure 2:**
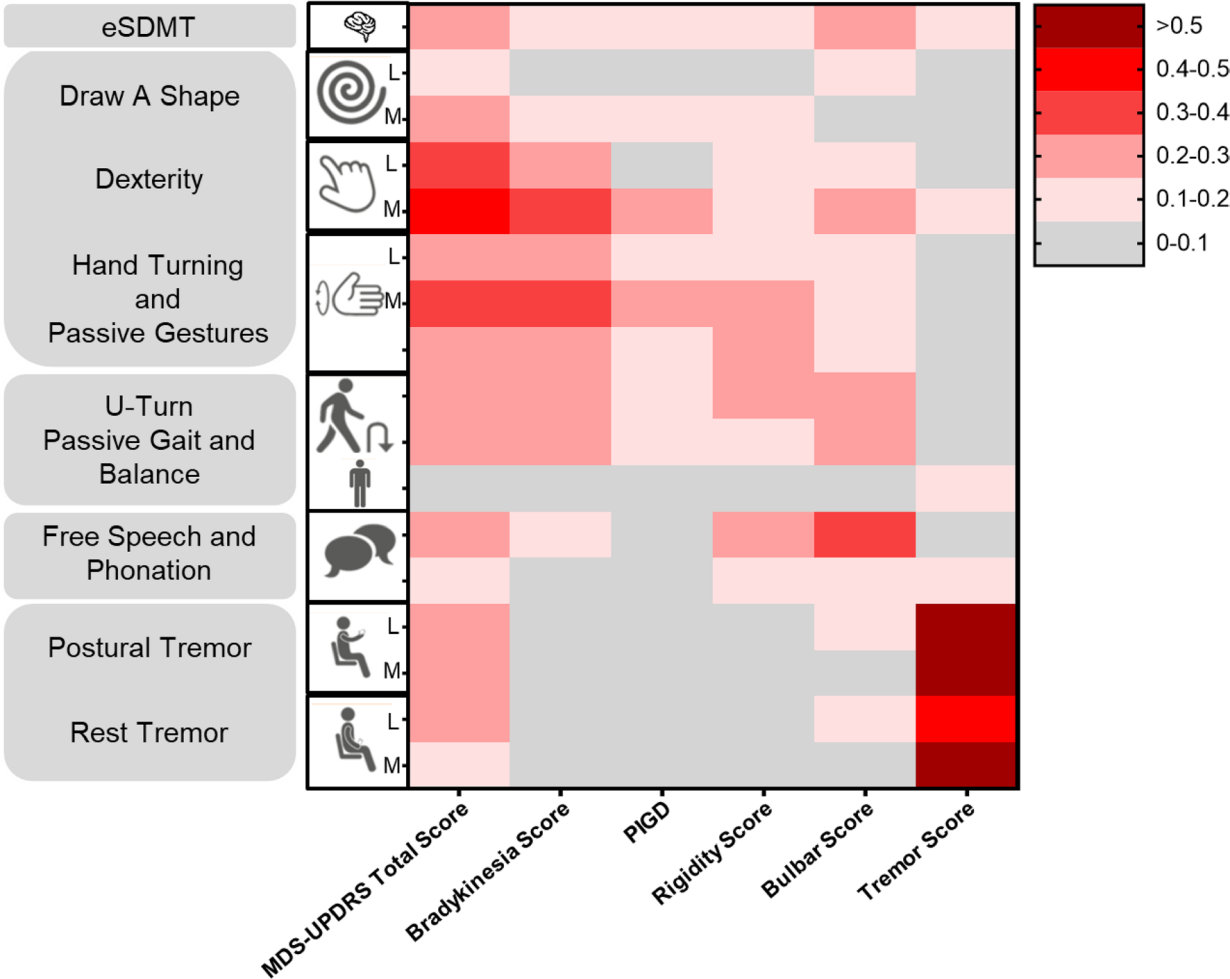
Absolute Spearman’s correlations between baseline MDS-UPDRS Total and Subscores and Roche PD Mobile Application v2 active test and passive monitoring sensor features. *eSDMT* electronic Symbol Digit Modalities Test, *MDS-UPDRS* Movement Disorder Society—Unified Parkinson's Disease Rating Scale, *PD* Parkinson’s disease, *PIGD* Postural Instability/Gait Disorders.

Cross-correlations between sensor features and MDS-UPDRS subscale scores demonstrated convergent and divergent validity of bradykinesia and tremor sensor features, with the strongest relationships between bradykinesia sensor features and MDS-UPRS subscores, and between tremor sensor features and MDS-UPDRS subscores, compared with all other subscores (Fig. 2). The Postural Instability/Gait Disorders (PIGD) subscale score showed the strongest correlations with the U-turn and the Hand Turning test (most affected side). Rigidity subscale scores correlated weakly with all sensor features overall, showing a weak-to-moderate relationship with Hand Turning in the most affected side and U-turn speed. The novel ‘bulbar score’ (defined in the methods section below) correlated most strongly with the Speech Test sensor feature. Overall, MDS-UPDRS total scores were most strongly related to bradykinesia sensor features, as expected for this early-stage population^1^.

### Sensor feature sensitivity to early disease manifestations and Hoehn and Yahr stage

**Figure 3:**
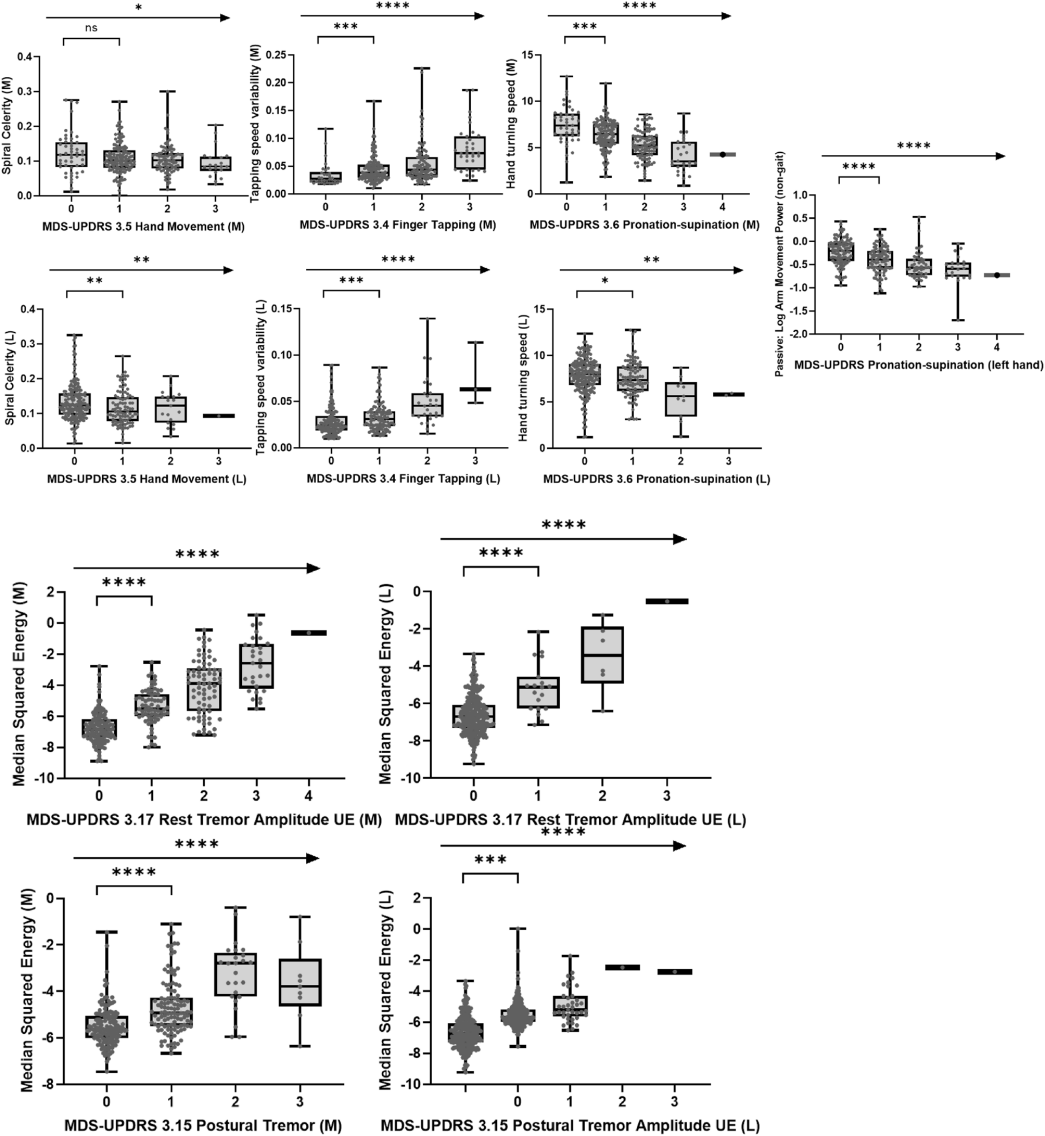
Association of sensor features from upper limb bradykinesia tests and upper limb tremor tests with corresponding clinical MDS-UPDRS measures at baseline (ns = *P* > 0.05; * = *P* ≤ 0.05; ** = *P* ≤ 0.01; *** = *P* ≤ 0.001; **** = *P* ≤ 0.0001). *L* less affected side, *M* more affected side, *MDS-UPDRS* Movement Disorder Society—Unified Parkinson's Disease Rating Scale, *UE* Upper Extremity.

**Figure 4:**
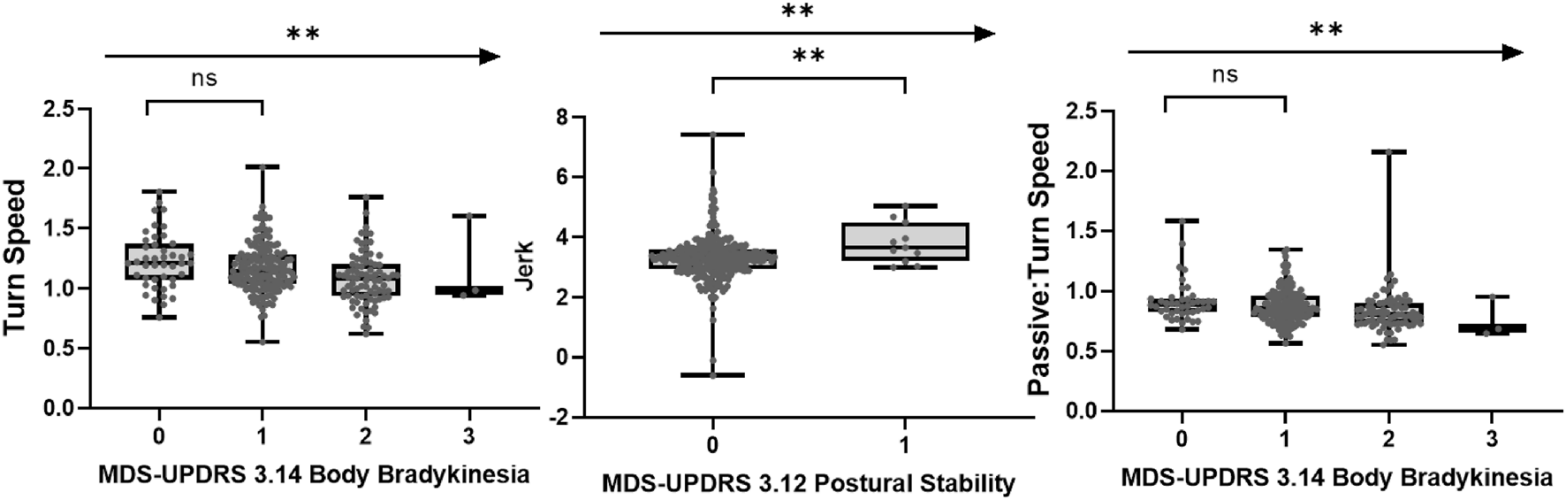
Association of sensor features from Phonation/Speech and eSDMT tests with corresponding clinical MDS-UPDRS measures at baseline (ns = *P* > 0.05; * = *P* ≤ 0.05; ** = *P* ≤ 0.01; *** = *P* ≤ 0.001; **** = *P* ≤ 0.0001). *eSDMT* electronic Symbol Digit Modalities test, *MDS-UPDRS* Movement Disorder Society—Unified Parkinson's Disease Rating Scale, *MFCC2* Mel Frequency Cepstral Coefficient 2.

**Figure 5:**
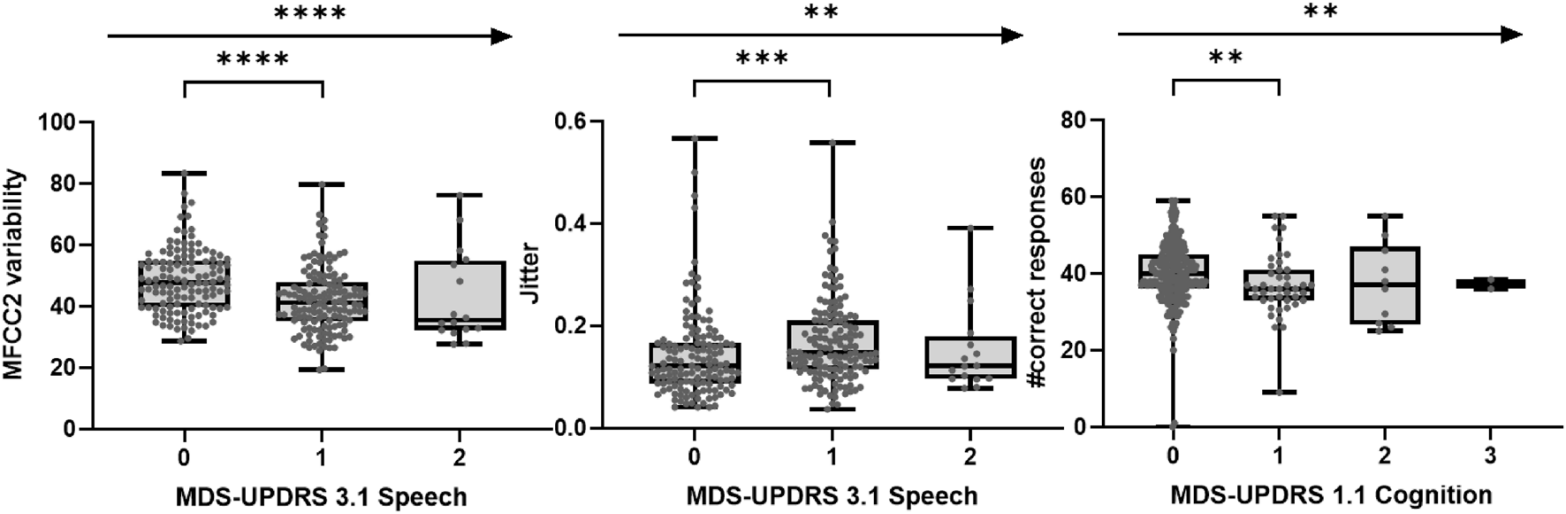
Association of sensor features from U-turn, Balance tests and Passive monitoring of gait with corresponding clinical MDS-UPDRS measures at baseline (ns = *P* > 0.05; * = *P* ≤ 0.05; ** = *P* ≤ 0.01; *** = *P* ≤ 0.001; **** = *P* ≤ 0.0001). *MDS-UPDRS* Movement Disorder Society—Unified Parkinson's Disease Rating Scale.

A total of 15/17 sensor features discriminated participants with scores of 0 versus 1 on associated MDS-UPDRS items (Table 1 and Figs. 3, 4, 5). Only sensor features from the Draw a Shape and U-turn tests showed borderline non-significant results. Participants in Hoehn and Yahr Stage I versus II were differentiated by 13 sensor features, i.e. all but Phonation test, Postural and Rest Tremor (most affected side) and the Balance test sensor features (Table 1).

### Sensor feature sensitivity to side differences

**Figure 6:**
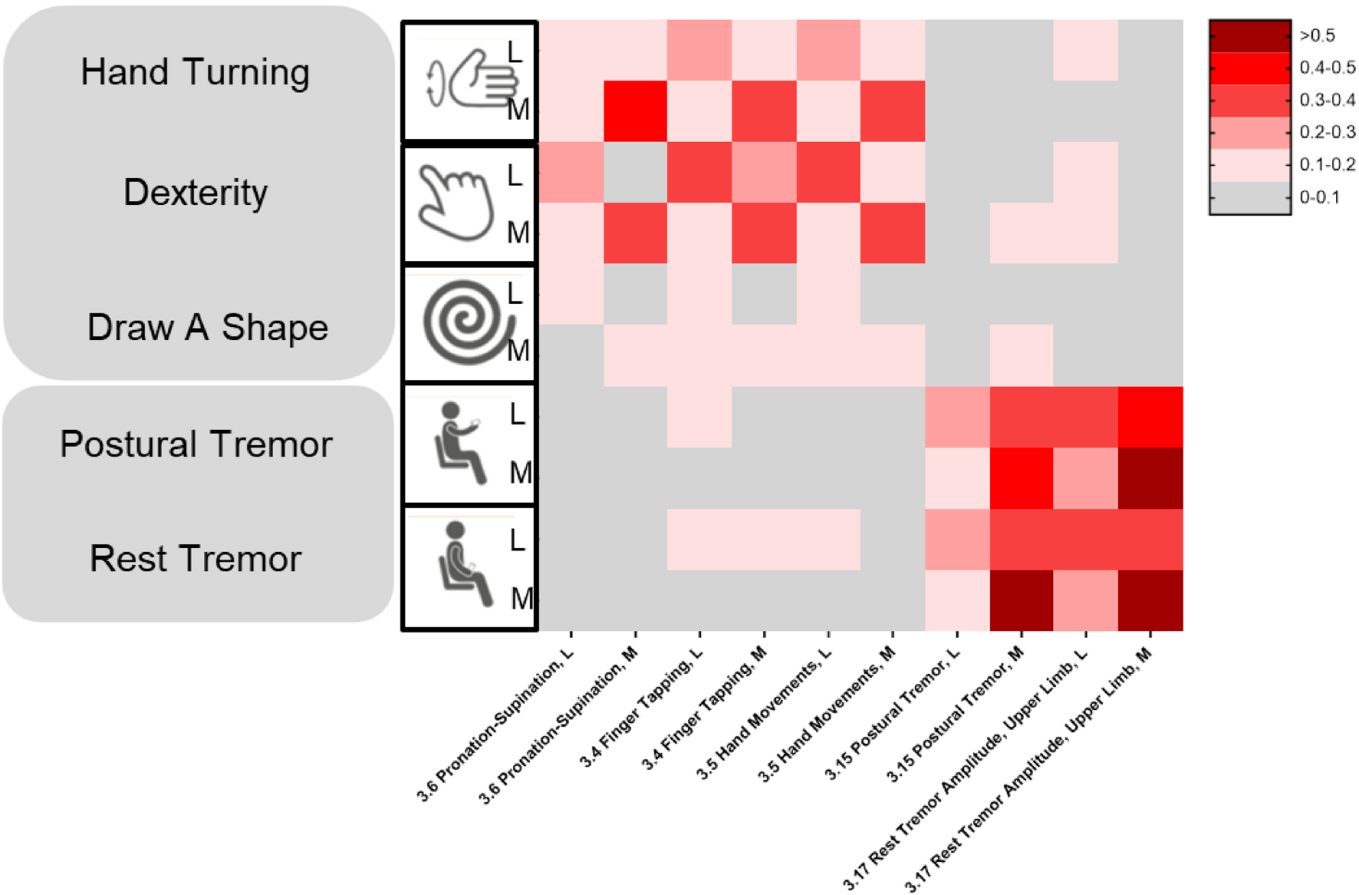
Correlations between active test sensor features from the more and less affected sides (M and L, respectively) with MDS-UPDRS Part III item scores evaluating M and L. *L* less affected side, *M* more affected side, *MDS-UPDRS* Movement Disorder Society—Unified Parkinson's Disease Rating Scale.

Sensor features values from all lateralized tests demonstrated significant differences between the most and least affected sides (Supplementary Table 1). Moreover, sensor features and MDS-UPDRS scores measuring the same motor sign on the less affected (or more affected) side were more strongly correlated than sensor features and MDS-UPDRS scores measuring the same motor sign on opposite sides of the body (Fig. 6).

## Discussion

### Reliability and validity of the Roche PD Mobile Application v2

The Roche PD Mobile Application v1 was designed to measure the core motor signs of PD^5,18^, and was recently revised to v2 to primarily include two new active tests of bradykinesia (Hand Turning, Draw A Shape), as well as a test of psychomotor slowing (eSDMT) and a speech test. In addition, the original gait task was revised to a U-turn test, and a smartwatch was incorporated into the remote passive monitoring procedure. Preliminary test–retest reliability scores for the pre-specified sensor features from all active tests except Speech and eSDMT, and for both passive monitoring measures, were in the ‘excellent’ range^22^. Preliminary clinical validity was established via correlations with corresponding MDS-UPDRS item scores. We note that these findings are reassuring considering the continuous (sensor feature) versus ordinal (MDS-UPDRS) nature of the two datasets, and the lack of conceptually comparable MDS-UPDRS items for some active test features (e.g. Draw A Shape). Cross-correlations between sensor features and MDS-UPDRS subscale scores supported the convergent and divergent validity of bradykinesia and tremor sensor features. Most active test sensor features demonstrated sensitivity for subtle manifestations, discriminating individuals who received MDS-UPDRS item scores of 0 from those with item scores of 1. Measures of upper limb bradykinesia demonstrated known-groups validity, differentiating individuals in Hoehn and Yahr Stage I versus II. All lateralized sensor features discriminated least versus the most affected sides of the body. The results from shared active tests and passive sensor features confirm previous findings with the Roche PD Mobile Application v1^5^. Taken together, these results indicate that the Roche PD Mobile Application v2 may prove suitable for quantifying motor disease severity and tracking disease progression in the earlier stages of PD.

### DHT measurement of bradykinesia

The Roche PD Mobile Application v2 contains three active tests designed to measure upper limb bradykinesia: Dexterity (finger tapping), Hand Turning (pronation/supination), and Draw A Shape. Pre-specified sensor features from all three tests correlated with their corresponding MDS-UPDRS upper limb bradykinesia item scores, and showed convergent and divergent validity in cross-correlations with MDS-UPDRS Part III subscale scores, correlating numerically most strongly with bradykinesia compared with all other subscale scores. These findings indicate that the Roche PD Mobile Application v2 bradykinesia tests indeed reflect the neurological concept of upper limb bradykinesia. Finger tapping and pronation/supination tasks are well-established assessments of upper limb bradykinesia as evidenced by their inclusion in both the UPDRS^24^ and MDS-UPDRS^8^. Over the last decade, different digitized variants of finger tapping and pronation/supination tests have been developed^21^. Despite methodological differences, studies of these DHT tasks generally showed good correspondence between finger tapping sensor features and respective clinical ratings, as well as the ability to differentiate healthy controls from individuals with early PD, and individuals with early PD from individuals with later-stage PD^5,18,25–28^, in line with the present findings. While the literature on digitized pronation/supination assessments is less rich than for finger tapping, available results also consistently demonstrate correlations with related clinical scores and the ability to differentiate healthy participants from individuals with PD^16,23,29–31^. Spiral drawing is traditionally used in behavioral neurology to assess fine motor impairment including bradykinesia and tremor^32–35^. DHT versions of spiral drawing demonstrated that time to completion correlated with clinician ratings of bradykinesia severity, and differentiated PD cases from controls^34^. The majority of previous DHT spiral drawing tasks used pens/digital pens to draw on regular paper or tablets, a more challenging motor task compared with the present finger drawing on smaller smartphone touch screens. In the present study, celerity, i.e. accuracy/time to complete spiral shape tracing on the smartphone screen, was pre-specified to additionally consider the accuracy of directed fine motor movements in the unsupervised at-home setting. Spiral celerity correlated with MDS-UPDRS bradykinesia measures, and the strength of these correlations was numerically smaller compared with Finger Tapping and Hand Turning. This may be due to the relative difficulty of the latter two tasks compared with spiral drawing, which may have challenged individuals more, thereby revealing greater impairment. We note that additional sensor features (e.g. variability in drawing speed, hesitation), analyzed either individually or combined within and across shapes, are expected to provide additional meaningful information, as has been shown for PD and multiple sclerosis^36,37^.

### Passive monitoring with smartwatches

Passive monitoring with smartwatches provides a unique opportunity to explore slowing of upper limb movements during daily life. Here, sensor data segments during arm movements were identified from the circa 90% non-walking periods in the passive monitoring sensor data stream, using the squared magnitude of the accelerometer sensor movement as the sensor feature. This same feature has been related to decreased expressivity in patients with schizophrenia with negative symptoms^38^. Here, arm movement power was specifically related to the MDS-UPDRS bradykinesia subscore and item scores, as well as the rigidity subscore, and is in line with a slowing of hand movement in daily non-gait-related activities such as gesturing when speaking, eating, etc. These findings are consistent with previous research with wrist-worn wearables, which traditionally focused on arm swing during gait^39–41^, as well as multi-sensor systems used to measure the impact of bradykinesia on activities of daily living^15,42^. Thus, passively monitored motor behavior in daily life may facilitate our understanding of the effect and burden of PD on individuals’ daily lives.

### DHT measurement of bradyphrenia

The eSDMT^43^ is commonly applied to measure psychomotor slowing, or bradyphrenia, one of the earliest cognitive signs in PD, appearing up to 5 years prior to a PD dementia diagnosis^20^. However, as the test requires multiple cognitive functions, it is not surprising that it is sensitive to many forms of neurologic impairment^44^. Indeed, while SDMT performance is reduced in PD^45^, impairments are exacerbated in individuals with PD with concomitant vascular^46^ and amyloid^47^ imaging findings. A standard SDMT outcome measure, number of correct responses in 90 s, was pre-specified for the present analyses of the eSDMT, and showed ‘good’^22^ test–retest reliability (ICC = 0.75). However, it correlated only weakly (rho = −0.18) with the MDS-UPDRS item 1.1. assessing global cognitive impairment. This finding is surprising given the catch-all nature of both the eSDMT and MDS-UPDRS item 1.1., but may be accounted for by the fact that cognitive impairments were excluded during the screening process in the PASADENA study, leading to a truncation of range in both scores (see Supplementary Fig. 1). We note that we attempted to minimize the effect of bradykinesia on eSDMT scores by requiring a simple tap response on a number pad displayed at the bottom half of the smartphone screen. Nevertheless, to mitigate the risk of this confound, eSDMT performance could be controlled by a non-cognitively demanding motor test using a similar response format.

### DHT measurement of voice and speech

Voice and speech impairments in PD are varied and generally summarized under the term dysarthria, and include resonatory, articulatory, phonatory, prosodic and respiratory components^48^. This symptomatology and its relevance to patients’ daily lives motivated the inclusion of a Sustained Phonation task in the suite of active tests, and the development of the novel Speech test. Voice jitter was pre-selected as a proxy of disordered vocal fold function for the sustained phonation test. In line with previous research^49^, increased voice jitter correlated weakly with MDS-UPDRS 3.1. (Speech) scores, and differentiated individuals with slight speech disturbances (MDS-UPDRS 3.1. score of 1) from those with no perceivable speech impairment at the site visit (MDS-UPDRS 3.1. score of 0). In the Speech active test, monotonicity (i.e. Mel Frequency Cepstral Coefficient 2 [MFCC] 2 fundamental frequency variability) was selected as the sensor feature of prosodic deficits based on previous research demonstrating that this feature differentiated individuals with PD from healthy controls^48^. In the present study, MFCC2 variability correlated with MDS-UPDRS 3.1. (Speech) scores, and differentiated participants with MDS-UPDRS 3.1. scores of 0 and 1. The bulbar MDS-UPDRS Part III composite item score was designed to gauge the severity of motor impairments in body parts involved in speech production. Despite a truncation of range in this score (average < 3/20 points), MFCC2 variability correlated with the bulbar score, indicating that this feature may estimate the severity of motor impairments in the speech apparatus. Future research will investigate further richly multi-faceted aspects of speech function to better understand motor and cognitive behavior in PD.

### DHT measurement of tremor, turning and balance

The Roche PD Mobile Application v2 aims to assess the broad array of motor signs in PD and related movement disorders. Thus, besides bradykinesia, speech, voice, and psychomotor slowing, tremor (rest, postural), turning during gait, and balance were also assessed. The rest and postural tremor active test features corresponded most strongly to the respective MDS-UPDRS concepts of tremor, as demonstrated by the highest correlation overall with any MDS-UPDRS item and subscale scores. This is consistent with similar DHT reports^5,25,50^. The novel U-turn test (which instructed individuals, if safe to do so, to walk several paces and make a U-turn at least five times) and the identification of turning while walking throughout the day in passive monitoring sensor data, were motivated by findings that turning is particularly impaired in PD^5,51,52^. For example, a 360 degree walking turn and instrumented timed-up-and-go test showed strong reliability and discriminated controls from PD participants^53,54^. Similarly, sensor-based measures of turn speed in daily life differentiated PD individuals from controls^55^. In the present study, turn speed measured in both the active test and passive setting correlated with MDS-UPDRS 3.14. body bradykinesia item scores, but was not specifically related to MDS-UPDRS PIGD relative to other subscores. While neither measure of turn speed differentiated between less and more affected individuals on MDS-UPDRS body bradykinesia scores of 0 versus 1, both differentiated between individuals in Hoehn and Yahr Stage I versus II. Although participants were not instructed to ‘turn as fast as possible’ to ensure a safe conduct of the active test, the U-turn test showed numerically higher correlations with body bradykinesia compared with passive turning speed, in line with similar profile of performance (active testing) versus capacity (passive monitoring) scores previously demonstrated for gait speed^56^. In the balance active test, the jerk sensor feature correlated with the MDS-UPDRS 3.12. postural stability item score, similar to previous reports^5,57^, and differentiated individuals with MDS-UPDRS item 3.12 scores of 0 versus 1, but failed to differentiate individuals in Hoehn and Yahr Stage I versus II. We speculate that this negative finding may reflect the low levels of gait and postural instability impairments in the present cohort (mean PIGD = 1).

### DHT composite scores

A composite summary score of individual features across diverse assessments is expected to provide a more robust measure of global PD severity and progression, especially given the heterogeneous nature of PD. Several DHT solutions besides the Roche PD Mobile Application v2 administer different motor active tests, and some additionally collect passive monitoring data^5,17,18^. Supplementary Table 2 provides a high-level comparison of these DHT solutions. All solutions contain active tests for tremor and tapping, but vary with respect to the inclusion of other upper limb, postural stability/gait, cognition, and voice/speech tests, and whether passively monitored motor data are collected. The power of combining different features across the tests in these DHTs has been shown via machine learning models that predict MDS-UPDRS total scores (Roche PD Mobile Application v1)^58^ or lead to a new score based on differentiation of ON and OFF L-dopa states^59^, and distinguished between healthy controls, idiopathic Rapid Eye Movement and PD^16,60^. A machine learning approach was also used to combine different HopkinsPD baseline sensor features to predict clinically significant events (e.g. falls, functional impairment) at the 18-month follow-up^61^. In contrast to data-driven approaches to composite score development, a clinical outcomes assessment approach could be applied whereby information from individuals with PD informs the selection of sensor features such that they optimally reflect what matters most to patients^62^.

### Limitations

Several facets of the present study limit the generalizability of the findings. Firstly, all individuals’ disease duration was < 2 years, and individuals were in Hoehn and Yahr Stages I or II. Thus, the applicability of the present findings to later-stage or prodromal PD is unknown. The reduced range of disease severities also appeared to limit the ranges of some DHT and clinical measures, which consequently limited the possibility to detect relationships between the two (Supplementary Fig. 1). Also, further research is necessary to better understand the suitability of this remote monitoring approach for later-stage patients with more severe cognitive or visual impairments. Second, since Roche PD Mobile Application v2 data are not yet available from neurologically normal individuals, sensor feature cut-off values differentiating normal from impaired motor behavior could not yet be calculated. It should be also noted that comparisons between DHT measures and clinical measures such as the MDS-UPDRS can also be affected by limitations in the clinical measures; if an active test is not adequately reflected by a clinical measure, the ability to detect meaningful correlations is reduced. Finally, only two continuous 2-week periods of DHT data were analyzed; thus, the long-term adherence to the remote monitoring procedure and ability of sensor features to detect changes over time remain to be established. Towards this end, it is critical to quantify and report test–retest reliabilities of sensor feature scores towards assessing a sensor feature’s potential to detect changes over time^63^ and any deviation from normal progression as a function of e.g. pharmacological interventions.

The Roche PD Mobile Application v2 was designed to measure the severity of early PD core motor signs and to provide information complementary to established clinical outcome measures. This remote monitoring approach enables high-frequency (i.e. daily) assessments with low average daily burden. The frequent measurement coupled with the high sensitivity of smartphone/smartwatch sensors may increase signal-to-noise of digital outcome measures for clinical research and provide novel insights into patients’ functioning in daily life.

## Methods

### Participants

Baseline Roche PD Mobile Application v2 data from 316 dopaminergic-treatment-naïve individuals recently diagnosed with dopamine transporter imaging with single-photon emission computed tomography-confirmed PD (Hoehn and Yahr Stages I–II, diagnosis ≤ 2 years) were analyzed (see Table 2 for demographic and clinical characteristics, and Supplementary Table 6 for non-parametric descriptive statistics). All individuals were enrolled in an ongoing randomized, double-blind, placebo-controlled, Phase II clinical trial (PASADENA Part 1; NCT03100149) of prasinezumab (RO7046015/PRX002), an anti-α-synuclein monoclonal antibody (see Pagano et al. 2021)^64^.

**Table 2:** Baseline characteristics of participants.

Characteristic	Participants (N = 316)
Age, mean (SD), years	59.9 (9.10)
Male, n (%)	213 (67%)
MDS-UPDRS Part IA Non-Motor Aspects of Experience of Daily Living (rater), mean (SD)	1.16 (1.62)
MDS-UPDRS Part IB Non-Motor Aspects of Experience of Daily Living (patient/caregiver), mean (SD)	3.45 (2.78)
MDS-UPDRS Part II Motor Aspects of Experiences of Daily Living (patient), mean (SD)	5.33 (4.04)
MDS-UPDRS Part III Motor Examination (rater), mean (SD)	21.47 (9.00)
MDS-UPDRS Part VI Motor Complications, mean (SD)	NA
MDS-UPDRS Total Score, mean (SD)	31.41 (12.78)
PIGD score, mean (SD)	1.04 (0.90)
Bradykinesia score, mean (SD)	10.50 (5.71)
Rigidity score, mean (SD)	4.58 (2.95)
Tremor score, mean (SD)	5.56 (4.01)
Speech score, mean (SD)	2.72 (2.19)
Axial symptoms, mean (SD)	2.04 (1.71)
**Hoehn and Yahr stage, %**	
Stage I	25
Stage II	75
Time since diagnosis, mean (SD), months	10.1 (6.5)
MoCA, mean (SD)	29.19 (1.01)

*MDS-UPDRS* Movement Disorder Society—Unified Parkinson's Disease Rating Scale, *MoCA* Montreal Cognitive Assessment, *NA* not applicable, *PIGD* Postural Instability/Gait Disorders, *SD* standard deviation.

All of the 59 PASADENA sites received approval from their institutional review boards or ethics committees and collected data used for the present analyses, and written informed consent was provided by all participants. The study is being conducted in accordance with the Declaration of Helsinki and the International Conference on Harmonization Guidelines for Good Clinical Practice.

### Roche PD Mobile Application v2

The Roche PD Mobile Application v2 consists of dedicated applications installed on a provisioned smartphone and smartwatch (see Fig. 1). The PD Mobile Application prompted participants to perform the active tests described below. All unilateral tests were performed twice, once with each side of the body.

1. *Draw A Shape*^37^ participants were instructed to trace six different shapes (two diagonal lines [once drawn up, once drawn downwards], and a square, circle, figure of 8, and spiral) on the smartphone screen with their index finger, as quickly and as accurately as possible (timeout: 30 s);
2. *Dexterity* participants were instructed to alternately tap two touchscreen buttons with their index finger as quickly and regularly as possible (20 s per hand);
3. *Hand Turning* holding the smartphone in the outstretched hand while seated, participants were instructed to rotate the hand as quickly and fully as possible such that the phone faced up and down (10 s per hand);
4. *Speech* participants were provided with three open-ended questions, one after the other, and instructed to read each out loud and answer each question out loud (20 s per question);
5. *Phonation* participants were instructed to make a single, continuous “aaaah” sound for as long as possible with one breath and in a steady pitch and volume while the phone was held at the ear (timeout: 30 s);
6. *Postural tremor* participants were instructed to sit with their eyes closed, and to hold the smartphone in an outstretched hand while counting down out loud from a pre-specified number that differed for each test administration (15 s per hand);
7. *Rest tremor* participants were instructed to sit with their eyes closed and to hold the phone in the palm of their hand, with their forearm resting on their thigh, and to count down out loud from a pre-specified number that differed for each test administration (15 s per hand);
8. *Balance* while standing with the smartphone in a running belt at waist height with the phone placed at the front of the body, participants were instructed to stand still with their arms at their side (30 s);
9. *U-turn* participants were instructed to place the smartphone in a running belt with the phone placed at the front of the body, and to walk between two points at least four steps apart at normal speed, completing at least five turns in 60 s;
10. *eSDMT*^43^ participants were instructed to match a sequence of displayed symbols to the respective numbers using a displayed coding key as quickly and as accurately as possible (90 s).

The Roche PD Mobile Application v2 additionally administered questionnaires, which are not the focus of the present report. For passive monitoring, participants were instructed to carry their smartphone (e.g. in their trouser pocket or in the pouch of a provided running belt) and wear their smartwatch as they conducted the daily active tests and their normal daily activities. No active tests were administered directly via the smartwatch.

### Procedure

Participants were provided with an Android smartphone (Galaxy S7, Samsung, Seoul, South Korea) and smartwatch (Moto G 360 2nd Gen Sport; Motorola, Chicago, USA) during a screening visit at the latest 7 days prior to the baseline clinical visit, and trained on the use of the devices and the Roche PD Mobile Application v2. Participants were instructed to open the application on the provisioned smartphone every morning. Active tests were scheduled automatically such that half of the motor tests were presented on alternating days, and the eSDMT every 2 weeks (Fig. 1), with a total expected testing time (per day) including transitions and test-start countdowns between tests of 5–10 min (including eSDMT). All data were stored in encrypted files on the smartphone and sent by WiFi to a cloud storage facility each time the smartphone connected to the Internet.

Baseline clinical assessments included the MDS-UPDRS^8^, from which subscale scores were generated (i.e. PIGD, bradykinesia, rigidity, tremor)^28^ The MDS-UPDRS was administered according to standardized procedures, and all MDS-UPDRS raters completed online MDS-UPDRS training by the MDS. Additionally, a ‘bulbar score’ was defined as the composite sum of MDS-UPDRS items 2.1 Speech; 2.2 Saliva and Drooling; 2.3 Chewing and Swallowing; 3.1 Speech; and 3.2 Facial expression.

### Sensor data processing

The raw sensor data from the smartphone and smartwatch were extracted and processed using a dedicated internally developed backend infrastructure. Custom algorithms implemented in Python were applied on quality-controlled sensor data (e.g. for correct test execution) and converted data into pre-defined ‘sensor features’, one per active test performed and side of body (if applicable) and one for passive monitoring. Features were selected based on previous literature and their relevance to PD (Supplementary Table 3).

1. *Draw a shape*: The feature *Spiral celerity* combines drawing accuracy and drawing speed (accuracy/speed) of the spiral drawing.
2. *Dexterity*: *Tapping variability* quantifies the variability of tapping speed as measured by the standard deviation of the time between consecutive tap events. This feature has already shown positive results in a previous study in PD^5^.
3. *Hand turning*: *Median hand turning speed* is the median turn speed over all segmented hand rotations to estimate bradykinesia.
4. *Speech*: The MFCC2 is the ratio between vocal tract resonation of the high and vocal fold vibration of the low Mel-frequency bands affected in PD. MFCC values have already shown case/control differences in other studies of PD^5^. For this novel feature, MFCC2s of consecutively voiced speech segments (i.e. parts of speech that are longer than 200 ms and are acoustically distinguishable) were calculated and averaged over each segment. MFCC2s are interpreted as a measure of speech monotonicity.
5. *Phonation*: *Voice jitter* is defined as a mean of the absolute differences between the period of adjacent pitch cycles, normalized by the mean pitch period, multiplied by 100. This definition of jitter is also referred to as jitter:local^49^. Jitter represents the variability of the speech fundamental frequency (pitch period) from one cycle to another and is a measure of micro-instability of vocal fold vibration, where higher values of the feature indicate higher instability of vocal fold vibration.
6. *Rest and postural tremor*: *Log median squared energy* measures the average acceleration magnitude, which is a proxy for the average amplitude during tremor induced by hand movements when trying to hold the hand still. A similar feature showed clinical validity for PD in a previous study^5^.
7. *Balance*: *Log sway jerk* describes the jerkiness (i.e. irregular, non-smooth accelerations) of movements when trying to stand still and may be a marker of disease progression in PD^65^.
8. *U-turn*: *Median turn speed* describes the average turn speed of all turns completed during the U-turn test. The same feature correlated with clinically assessed gait impairment in previous studies in individuals with PD and multiple sclerosis^5^.
9. *SDMT*: *Number of correct responses* is the standard feature also reported in the traditional paper-based in-clinic SDMT^43^.
10. *Passive monitoring—gait*: *Median turn speed in passive monitoring* is the average turn speed of all turns detected over a given day of smartphone sensor recording, and had previously demonstrated discriminability between individuals with PD and control participants^66^.
11. *Passive monitoring—non-gait arm movements*^67^: *Median arm movement power (non-gait)* is the median of the integrated squared acceleration magnitude (i.e. power) over all identified arm movements during non-gait data segments in a given day of sensor recording with the smartwatch. As such, it measures the intensity of arm movements during activities of daily living (gesturing when speaking, grabbing something, etc.) that do not occur during periods of walking (i.e. does not reflect arm swing while walking). Here, we hypothesize that a reduced intensity of arm movements is associated with bradykinesia.

All features reported in this manuscript are based on smartphone sensor data, with the exception of ‘arm movement power (non-gait)’, which leverages passively acquired sensor data collected with the smartwatch.

Data underwent quality control (QC) checks to ensure that the tests had been performed properly. Towards this end, QC metrics were generated. For example, one QC metric quantified the amount of energy from the accelerometer during the Hand Turning test to estimate whether the smartphone was lying still (e.g. on a table) or moving during the test. 0.3% (n = 179/56,786) of digital active test data did not meet the pre-specified QC thresholds and were therefore excluded from the analyses.

### Statistical analyses

Sensor features from passive monitoring and each active test performed were summarized (median) over 2-week intervals starting at the baseline visit (Weeks 1 and 2) and in the 2-week period thereafter, provided that ≥ 3 data points were available during each 2-week testing interval. Where applicable, sensor features were assigned to less/more affected side (for definition see Supplementary Material). For convergent/divergent validity (i.e. degree of association with related/unrelated symptom domains), the averaged (median) sensor data collected during the first two study weeks were compared with clinical data collected at the baseline visit (Day 1) using Spearman’s correlations. Adherence and test–retest metrics were calculated for aggregated sensor features for the first two 2-week study periods. Adherence was defined as the number of fully completed active testing sessions relative to the number of all possible active testing sessions. For passive monitoring, also calculated over the first two 2-week study periods, the average number of hours per day participants carried the provisioned study smartphone with them and wore the study smartwatch was calculated. Sensor feature test–retest reliabilities were quantified with the ICC between averaged values of the first and second contiguous 2-week periods. To investigate the sensitivity of sensor features to subtle or very early symptoms, sensor features from participants receiving MDS-UPDRS item scores of 0 versus 1 were compared using Mann–Whitney U tests. For known-groups validity (i.e. differences between pre-defined groups where a difference is prima facie expected), sensor features were compared between participants in Hoehn and Yahr Stage I versus II, and by comparing sensor feature values from less and more affected sides, both using Mann–Whitney U tests.

### Ethics declarations

Participants were identified for potential recruitment using site-specific recruitment plans prior to consenting to take part in this study. Recruitment materials for participants had received Institutional Review Board or Ethics Committee approval prior to use. The following Institutional Review Boards ruled on ethics of the PASADENA study: Ethikkommission der Medizinischen Universität Innnsbruck, Innsbruck, Austria; Comité de Protection des Personnes (CPP) Ouest IV, Nantes, France; Ethikkommission der Universität Leipzig and Geschäftsstelle der Ethikkommission an der medizinischen Fakultät der Universität Leipzig, Leipzig, Germany; Ethikkommission der Fakultät für Medizin der Technischen Universität München, München, Germany; Ethikkommission der Universität Ulm (Oberer Eselsberg), Ulm, Germany; Landesamt für Gesundheit und Soziales Berlin and Geschäftsstelle der Ethik-Kommission des Landes Berlin, Berlin, Germany; Ethikkommission des FB Medizin der Philipps-Universität Marburg, Marburg, Germany; Ethikkommission an der Medizinischen Fakultät der Eberhard-Karls-Universität und am Universitätsklinikum Tübingen, Tübingen, Germany; Ethikkommission an der medizinischen Fakultät der HHU Düsseldorf, Düsseldorf, Germany; Ethikkommission der LÄK Hessen, Frankfurt, Germany; CEIm Hospital Universitari Vall d’Hebron, Barcelona, Spain; Copernicus Group Independent Review Board, Puyallup, Washington, USA; Western Institutional Review Board, Puyallup, Washington, USA; the University of Kansas Medical Center Human Research Protection Program, Kansas City, Kansas, USA; Oregon Health & Science University Independent Review Board, Portland, Oregon, USA; Northwestern University Institutional Review Board, Chicago, Illinois, USA; Spectrum Health Human Research Protection Program, Grand Rapids, Michigan, USA; the University of Vermont Committees on Human Subjects, Burlington, Vermont, USA; Beth Israel Deaconess Medical Center Committee on Clinical Investigations, New Procedures and New Forms of Therapy, Boston, Massachusetts, USA; Vanderbilt Human Research Protection Program Health Sciences, Nashville, Tennessee, USA; University of Maryland, Baltimore Institutional Review Board, Baltimore, Maryland, USA; University of Southern California Institutional Review Board, Los Angeles, California, USA; Columbia University Medical Center Institutional Review Board, New York, New York, USA; University of Southern California San Francisco Institutional Review Board, San Francisco, California, USA; University of Pennsylvania Institutional Review Board, Philadelphia, Philadelphia, USA; HCA—HealthOne Institutional Review Board, Denver, Colorado, USA. All Institutional Review Boards gave ethical approval of the study.

## Supplementary Information

Supplementary Information.

## Data Availability

Data will be made available upon reasonable request. Please direct your request to dbm.datarequest@roche.com. For further details on Roche's Global Policy on the Sharing of Clinical Information and how to request access to related clinical trial documents, see https://www.roche.com/research_and_development/who_we_are_how_we_work/clinical_trials/our_commitment_to_data_sharing.htm.

## Code availability

The code used to perform all of the statistical analyses described herein are available upon request to dbm.datarequest@roche.com.
