# Supplementary material for: Reliability and validity of the Roche PD Mobile Application for remote monitoring of early Parkinson’s disease
Source: Sci Rep. 2022 Jul 15;12:12081. doi: 10.1038/s41598-022-15874-4 (PMC9287320; doi:10.1038/s41598-022-15874-4)
Supplement: Supplementary file 1 — Supplementary Information. [file 41598_2022_15874_MOESM1_ESM.docx]

**Reliability and validity of the Roche PD Mobile Application for remote monitoring of early Parkinson’s disease**

Authors: Florian Lipsmeier*^†1^, Kirsten I. Taylor^†1^, Ronald B. Postuma^2^, Ekaterina
Volkova-Volkmar^1^, Timothy Kilchenmann^1^, Brit Mollenhauer^3,4^, Atieh Bamdadian^1^, Werner L. Popp^1^, Wei-Yi Cheng^1^, Yan Ping Zhang^1^, Detlef Wolf^1^, Jens Schjodt-Eriksen^1^, Anne Boulay^5^,
Hanno Svoboda^1^, Wagner Zago^6^, Gennaro Pagano^1^, Michael Lindemann^1^

^*^ Corresponding Author

^†^ equal contribution

1. Roche Pharma Research and Early Development, pRED Informatics, Pharmaceutical Sciences, Clinical Pharmacology, and Neuroscience and Rare Diseases Discovery and Translational Area, Roche Innovation Center Basel, F. Hoffmann-La Roche Ltd., Basel, Switzerland;
2. Department of Neurology, McGill University, Montreal General Hospital, Montreal, Quebec, Canada;
3. Paracelsus-Elena-Klinik, Kassel, Germany;
4. Department of Neurology, University Medical Center Göttingen, Göttingen, Germany;
5. Idorsia Pharmaceuticals Ltd, Allschwil, Switzerland;
6. Prothena Biosciences Inc, South San Francisco, CA, USA.

Supplementary materials

**Definition of less/more affected side:**

The most affected side was defined as the side of the body with the highest MDS-UPDRS Part III summed item score for all lateralized items. If the score for the right side of the body was greater than that for the left, then the right side of the body was defined as the most affected side, and vice versa. If both scores were equal at baseline, then the side of the body with the larger/smaller lateralized MDS-UPDRS Part III score from the screening visit was used to define the most/least affected side. If both lateralized scores from the screening visit were still equal, then the most affected side was defined as the side of the body contralateral to the most affected putamen based on DaT-SPECT Striatal Binding Ratios (i.e., the putamen with the lower SBR). Using this protocol, all 316 patients could be assigned a most and least affected side.

**Supplementary Table 1. Differences in sensor feature values from lateralized active tests between less affected and more affected sides. (green color indicates that the difference is in the expected direction). A Wilcoxon signed-rank test was used for statistical comparisons.**

| **Active test** | **W** | ***P* value** | **Percent difference** |
| --- | --- | --- | --- |
| Rest Tremor | 6304.0 | < 0.0001 | 12.97 |
| Postural Tremor | 10118.0 | < 0.0001 | 6.89 |
| Hand Turning | 2936.0 | < 0.0001 | –22.7 |
| Dexterity | 6083.0 | < 0.0001 | 50.78 |
| Draw A Shape | 11724.0 | < 0.0001 | –13.45 |

**Supplementary Table 2. Smartphone- and smartwatch-based active test suites which were tested in more than 30 participants with PD.**

| **Domain** | **Test** | **mPower** | **HopkinsPD** | **CloudUPDRS** | | **Roche v1*** | **Roche v2**** |
| --- | --- | --- | --- | --- | --- | --- | --- |
| Tremor upper limb | Rest Tremor |  | x | X | x | | x |
|  | Postural Tremor |  | x | X | x | | x |
|  | Kinetic Tremor |  |  | X |  | |  |
| Tremor lower limb | Rest Tremor |  |  | X |  | |  |
| Upper limb bradykinesia | Hand turning |  |  | X |  | | x |
|  | Finger tapping^1,2^ | x | x | X | x | | x |
|  | Draw A Shape^3^ |  |  |  |  | | x |
| Gait/postural instability | Balance |  | x |  | x | | x |
|  | U-turn |  |  |  |  | | x |
|  | Gait | x | x | X | x | | x |
|  | Leg agility |  |  | X |  | |  |
| Voice/Speech | Sustained phonation | x | x |  | x | | x |
|  | Speech |  |  |  |  | | x |
| Cognition | eSDMT^4^ |  |  |  |  | | x |
|  | Memory | x |  |  |  | |  |
|  | Reaction time |  | x |  |  | |  |
| Passive monitoring phone |  |  | x |  | x | | x |
| Passive monitoring watch |  |  |  |  |  | | x |

*** Roche PD Mobile Application v1.**

**** Roche PD Mobile Application v2.**

**eSDMT, electronic Symbol Digit Modalities test; PD, Parkinson’s disease; UPDRS, Unified Parkinson’s Disease Rating Scale.**

**Supplementary Fig. 1. Distributions of MDS-UPDRS parts and subscores at baseline clinical visit**


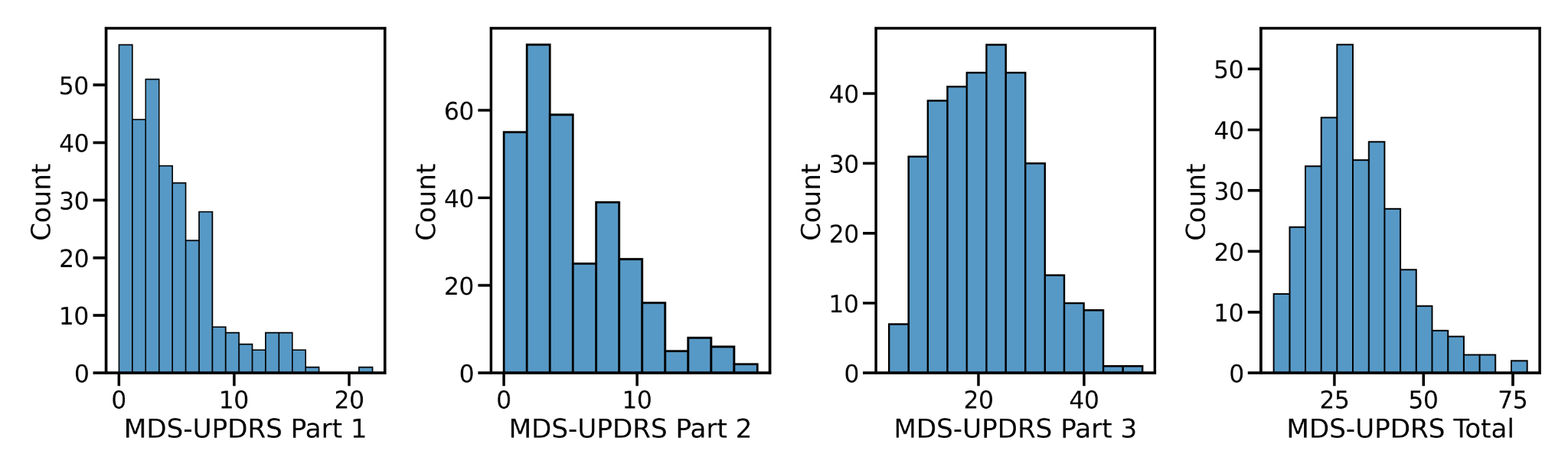


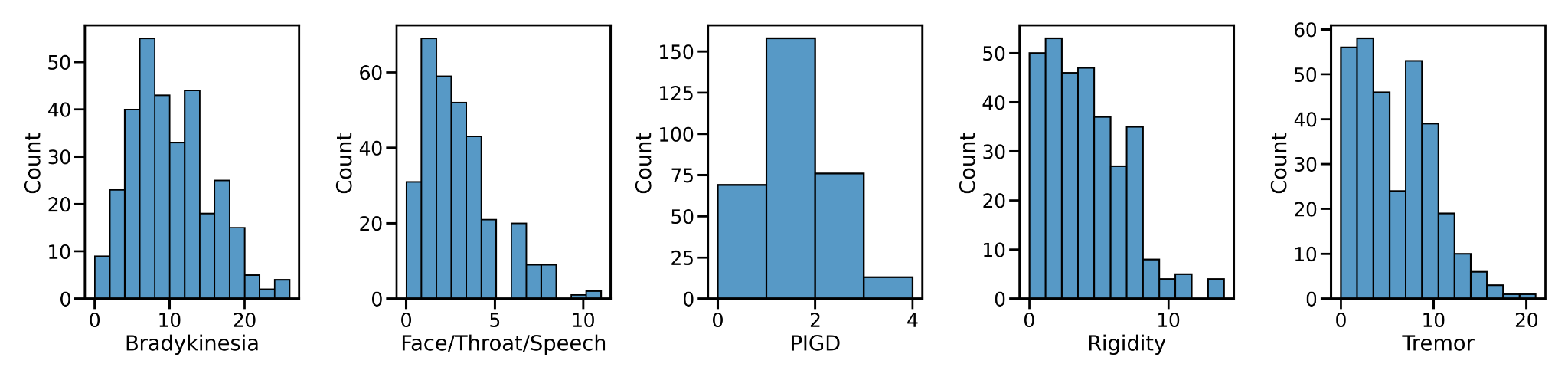


**MDS-UPDRS, Movement Disorder Society - Unified Parkinson's Disease Rating Scale; PIGD, Postural Instability/Gait Disorders.**

**Supplementary Table 3. Pre-specified sensor feature for each active test and for passive monitoring**

| **DHT assessment** | **Sensor feature (unit)** | **Description** |
| --- | --- | --- |
| Draw A Shape | Spiral celerity (1/sec) | Let $t_{f}$ be the time until the spiral drawing is finished and *a* be the accuracy of drawing, as defined by the percentage of the line that intersects with the pre-defined spiral, then *Spiral celerity* is defined as $\frac{a}{t_{f}}$ ^3^ |
| Dexterity | Tapping variability (sec) | Let $t_{1,}t_{2},\ldots,t_{n}$ be the time between consecutive left/right button taps, then *Tapping variability* is defined as $\sqrt{\frac{\sum(t_{i}-avg\left( t_{i} \right))}{n}}$ *^5^* |
| Hand Turning | Median hand turning speed (rad/s) | Let ${ts}_{1},\ldots,{ts}_{n}$be the average turn speed for a given hand turning segment as measured with the gyroscope, then *Median hand turning speed* is defined as $Median({ts}_{1},\ldots{ts}_{n})$ |
| Speech | MFCC2 variability (-) | Let $m_{1},m_{2},\ldots,m_{n}$ be the average MFCCC2s per consecutive voiced segment, then *MFCC2 variability* is defined as $Mean(\left\vert\left\{ m_{2}-m_{1},\ldots,m_{n}-m_{n-1} \right\} \right\vert)$ |
| Phonation | Voice jitter (%) | Let ${pc}_{1},\ldots,{pc}_{n}$be consecutive pitch cycles, *mp* the mean pitch period, then *Voice jitter* is $\frac{Mean\left( \left\vert\left\{ {pc}_{2}-{pc}_{1},\ldots,{pc}_{n}-{pc}_{n-1} \right\} \right\vert\right)}{mp}*100$ |
| Rest and postural tremor | Log median squared energy (m^2^/sec^4^) | Let $a={a_{x}}^{2}+{a_{y}}^{2}+{a_{z}}^{2}$ be the squared magnitude of acceleration and $a_{1,..,}a_{n}$be the consecutive acceleration magnitude signals, then *Log median squared energy* is $Log(Median\left( a_{1,..,}a_{n} \right))$ ^5^ |
| Balance | Log sway jerk (m^2^/s^5^) | See Mancini et al. ^6^ |
| U-turn | Median turn speed (rad/s) | Let ${ts}_{1},\ldots,{ts}_{n}$ be the average speeds of individual turns measured with the gyroscope, then *Median turn speed* is defined as $Median({ts}_{1},\ldots,{ts}_{n})$ ^7^ |
| SDMT | Number of correct responses | -- |
| Passive monitoring (smartphone, gait) | Median turn speed in passive monitoring (rad/s) | Let ${ts}_{1},\ldots,{ts}_{n}$ be the average speeds of individual turns measured with the gyroscope, then *Median turn speed* is defined as $Median({ts}_{1},\ldots,{ts}_{n})$ ^8^ |
| Passive monitoring (smartwatch, arm movements) | Median arm movement power (m^2^/se  c^3^) | Let $gp= \int{{(a}_{t}-mean\left( a_{t} \right))}^{2}dt$  be the arm movement power of an identified non-gait arm movement, then *Median arm movement power* is $Median({gp}_{1},..,{gp}_{n})$ ^9^ |

**MFCC2, Mel-frequency cepstral coefficient 2; SDMT, Symbol Digit Modalities Test.**

**Supplementary Table 4. Description of QC criteria when a test feature is excluded due to a test execution that is not in line with the test instructions**

| **DHT assessment** | **Sensor feature** | **Test exclusion QC description** |
| --- | --- | --- |
| Draw A Shape | Spiral celerity | Spiral is drawn with lower than 10% accuracy in less than 0.5 seconds |
| Dexterity | Tapping variability | No test-relevant screen interactions (finger down then up) are detected |
| Hand Turning | Median Hand Turning speed | The smartphone is on a stable horizontal surface (e.g. a table) during the test |
| Speech | MFCC2 variability | Fewer than 3 speech segments minimally 0.2 seconds) and (less than 0.2 seconds of voicing) |
| Phonation | Voice jitter | No voicing segments above 0.2 seconds in length |
| Rest and postural tremor | Log median squared energy | The smartphone is on a stable horizontal surface (e.g. a table) during the test |
| Balance | Log sway jerk | The smartphone is on a stable horizontal surface (e.g. a table) during the test |
| U-turn | Median turn speed | The smartphone is on a stable horizontal surface (e.g. a table) during the test |
| SDMT | Number of correct responses | Fast and inaccurate test execution. Response accuracy rate is below 20% and average response time is less than 1 second |
| Passive monitoring (smartphone, gait) | Median turn speed in passive monitoring | NA |
| Passive monitoring (smartwatch, gestures) | Median hand movement power | NA |

**MFCC2, Mel- frequency cepstral coefficient 2; NA, not applicable; QC, quality control; SDMT, Symbol Digit Modalities Test.**

**Supplementary Table 5. MDS-UPDRS group (item score 0 vs 1) and Hoehn and Yahr group (stage 1 vs 2) median sensor feature values.**

| **Digital test** | **Sensor feature** | **MDS-UPDRS item** | **MDS-UPDRS item score 0 vs 1** | | **Hoehn and Yahr Stage I vs II** | | |
| --- | --- | --- | --- | --- | --- | --- | --- |
|  |  |  | 0 group | 1 group | Stage I | Stage II | |
| **Draw A Shape** | Spiral celerity (L) | 3.5 Hand movement (L) | 0.1236 | 0.1062 | 0.126 | 0.1141 |  |
|  | Spiral celerity (M) | 3.5 Hand movement (M) | 0.118 | 0.1011 | 0.1174 | 0.0996 |  |
| **Dexterity** | Tapping variability (L) | 3.4 Finger tapping (L) | 0.0242 | 0.0308 | 0.0235 | 0.0299 |  |
|  | Tapping variability (M) | 3.4 Finger tapping (M) | 0.0271 | 0.0381 | 0.0371 | 0.0437 |  |
| **Hand Turning** | Median hand turning speed (L) | 3.6 Pronation-supination (L) | 7.9792 | 7.3529 | 8.2228 | 7.6327 |  |
|  | Median hand turning speed (M) | 3.6 Pronation-supination (M) | 7.3644 | 6.4536 | 6.4098 | 5.8119 |  |
| **Speech** | MFCC2 variability | 3.1 Speech | 47.3846 | 41.1341 | 45.2773 | 43.1431 |  |
| **Phonation** | Voice jitter | 3.1 Speech | 0.1215 | 0.1478 | 0.1255 | 0.1417 |  |
| **Postural Tremor** | Log median squared energy (L) | 3.15 Postural tremor (L) | -5.6713 | -5.203 | -5.6566 | -5.5886 |  |
|  | Log median squared energy (M) | 3.1 Postural tremor (M) | -5.6322 | -4.9308 | -5.2637 | -5.1964 |  |
| **Rest Tremor** | Log median squared energy (L) | 3.17 Rest tremor amplitude (L) | -6.708 | -5.1159 | -6.9799 | -6.5527 |  |
|  | Log median squared energy (M) | 3.17 Rest tremor amplitude (M) | -6.8546 | -5.5338 | -5.9101 | -5.6978 |  |
| **Balance** | Log sway jerk | 3.12 Postural stability | 3.3004 | 3.6501 | 3.2701 | 3.3124 |  |
| **U-turn** | Median turn speed | 3.14 Body bradykinesia | 1.2134 | 1.148 | 1.2097 | 1.1151 |  |
| **eSDMT** | Number of correct responses | 1.1 Cognitive impairment | 40 | 36 | 42 | 39 |  |
| **Passive monitoring of gait** | Median turn speed | 3.14 Body bradykinesia | 0.8839 | 0.8547 | 0.8779 | 0.837 |  |
| **Passive monitoring of gesture** | Median hand movement power | 3.6 Pronation-supination, left hand | 0.6013 | 0.4654 | 0.5488 | 0.3807 |  |

**eSDMT, electronic Symbol Digit Modalities Test; MDS-UPDRS, Movement Disorder Society - Unified Parkinson's Disease Rating Scale; MFCC2, Mel- frequency cepstral coefficient 2.**

**Supplementary Table 6. Non parametric descriptive statistics of PD patient (n=316) demographics and clinical characteristics.**

| **Characteristic** | **Median**  **(25th quantile, 75th quantile), N=316** |
| --- | --- |
| **Age, mean (SD), years** | 61.0 (53.0, 67.0) |
| **Male, n (%)** | 213 (67%) |
| **MDS-UPDRS Part IA Non-Motor Aspects of Experience of Daily Living (rater), mean (SD)** | 1.0 (0.0, 2.0) |
| **MDS-UPDRS Part IB Non-Motor Aspects of Experience of Daily Living (patient/caregiver), mean (SD)** | 3.0 (1.0, 5.0) |
| **MDS-UPDRS Part II Motor Aspects of Experiences of Daily Living (patient), mean (SD)** | 4.0 (2.0, 8.0) |
| **MDS-UPDRS Part III Motor Examination (rater), mean (SD)** | 21.0 (15.0, 28.0) |
| **MDS-UPDRS Part VI Motor Complications, mean (SD)** | NA |
| **MDS-UPDRS Total Score, mean (SD)** | 30.0 (23.0, 39.0) |
| **PIGD score, mean (SD)** | 1.0 (1.0, 2.0) |
| **Bradykinesia score, mean (SD)** | 9.0 (6.0, 13.0) |
| **Rigidity score, mean (SD)** | 4.0 (2.0, 6.0) |
| **Tremor score, mean (SD)** | 5.0 (2.0, 8.25) |
| **Speech score, mean (SD)** | 2.0 (1.0, 4.0) |
| **Axial symptoms, mean (SD)** | 2.0 (1.0, 3.0) |
| **Hoehn and Yahr stage, %  Stage I**  **Stage II** | 25  75 |
| **Time since diagnosis, mean (SD), months** | 8.50 (4.97, 13.52) |

**MDS-UPDRS, Movement Disorder Society - Unified Parkinson's Disease Rating Scale; PIGD, Postural Instability/Gait Disorders; SD, standard deviation.**

**References**

1 Movement Disorder Society Task Force on Rating Scales for Parkinson's Disease. The Unified Parkinson's Disease Rating Scale (UPDRS): Status and recommendations. *Movement disorders : official journal of the Movement Disorder Society* **18**, 738-750, doi:10.1002/mds.10473 (2003).

2 Goetz, C. G. *et al.* Movement Disorder Society-sponsored revision of the Unified Parkinson's Disease Rating Scale (MDS-UPDRS): scale presentation and clinimetric testing results. *Movement disorders : official journal of the Movement Disorder Society* **23**, 2129-2170, doi:10.1002/mds.22340 (2008).

3 Creagh, A. P. *et al.* Smartphone-based remote assessment of upper extremity function for multiple sclerosis using the Draw a Shape Test. *Physiol Meas* **41**, 054002, doi:10.1088/1361-6579/ab8771 (2020).

4 Johnson, D. K., Langford, Z., Garner-Villarreal, M., Morris, J. C. & Galvin, J. E. Onset of mild cognitive impairment in Parkinson disease. *Alzheimer Dis Assoc Disord* **30**, 127-133 (2016).

5 Lipsmeier, F. *et al.* Evaluation of smartphone-based testing to generate exploratory outcome measures in a phase 1 Parkinson's disease clinical trial. *Movement disorders : official journal of the Movement Disorder Society* **33**, 1287-1297, doi:10.1002/mds.27376 [doi] (2018).

6 Mancini, M. *et al.* ISway: a sensitive, valid and reliable measure of postural control. *J Neuroeng Rehabil* **9**, 59, doi:10.1186/1743-0003-9-59 (2012).

7 Cheng, W. Y. *et al.* U-turn speed is a valid and reliable smartphone-based measure of multiple sclerosis-related gait and balance impairment. *Gait & posture* **84**, 120-126, doi:10.1016/j.gaitpost.2020.11.025 (2021).

8 Cheng, W.-Y. *et al.* Large-Scale Continuous Mobility Monitoring of Parkinson’s Disease Patients Using Smartphones. *International Conference on Wireless Mobile Communication and Healthcare* 12-19, doi:10.1007/978-3-319-98551-0_2 (2018).

9 Umbricht, D., Cheng, W. Y., Lipsmeier, F., Bamdadian, A. & Lindemann, M. Deep learning-based human activity recognition for continuous activity and gesture monitoring for schizophrenia patients with negative symptoms. *Frontiers in Psychiatry* **11**, 574375, doi:10.3389/fpsyt.2020.574375 (2020).
